# Whether coagulation dysfunction influences the onset and progression of diabetic peripheral neuropathy: A multicenter study in middle‐aged and aged patients with type 2 diabetes

**DOI:** 10.1111/cns.70040

**Published:** 2024-09-11

**Authors:** Jiali Xie, Xinyue Yu, Luowei Chen, Yifan Cheng, Kezheng Li, Mengwan Song, Yinuo Chen, Fei Feng, Yunlei Cai, Shuting Tong, Yuqin Qian, Yiting Xu, Haiqin Zhang, Junjie Yang, Zirui Xu, Can Cui, Huan Yu, Binbin Deng

**Affiliations:** ^1^ Department of Neurology First Affiliated Hospital of Wenzhou Medical University Wenzhou P.R. China; ^2^ Department of Neurology, Shanghai East Hospital Tongji University School of Medicine Shanghai P.R. China; ^3^ Alberta Institute Wenzhou Medical University Wenzhou China; ^4^ Department of Neurology The Second Affiliated Hospital of Zhejiang University, School of Medicine Hangzhou China; ^5^ Department of Neurology Center for Rehabilitation Medicine Zhejiang Provincial People's Hospital, Affiliated People's Hospital, Hangzhou Medical College Hangzhou China; ^6^ Department of Neurology, First Clinical College of Wenzhou Medical University Wenzhou P.R. China; ^7^ Department of Neurology Ruian People's Hospital Wenzhou P.R. China; ^8^ Department of Neurology Shaoxing People's Hospital Shaoxing P.R. China; ^9^ Department of Neurology, Anyang District Hospital, Beiguan District Anyang Henan China; ^10^ Department of Neurology Institute of Neurology, Ruijin Hospital, Shanghai Jiao Tong University School of Medicine Shanghai China; ^11^ Institute of Environmental Medicine Karolinska Institutet Stockholm Sweden; ^12^ Department of Pediatrics Second Affiliated Hospital and Yuying Children's Hospital of Wenzhou Medical University Wenzhou P.R. China

**Keywords:** coagulation function, diabetic peripheral neuropathy, electromyography, type 2 diabetes

## Abstract

**Background:**

Nearly half of patients with diabetes experience diabetic peripheral neuropathy (DPN), resulting in a mere 53% survival rate within 3 years. Aberrations in coagulation function have been implicated in the pathogenesis of microvascular complications, prompting the need for a thorough investigation into its role as a contributing factor in the development and progression of DPN.

**Methods:**

Data were gathered from 1211 type 2 diabetes patients admitted to five centers from September 2018 to October 2022 in China. DPN was evaluated by symptoms and electromyography. Motor and sensory nerve conduction velocity (NCV) was appraised and the NCV sum score was calculated for the median, ulnar, and peroneal motor or sensory nerves.

**Results:**

Patients with DPN exhibited alterations in coagulation function. (i) Specifically, they exhibited prolonged thrombin time (*p* = 0.012), elevated fibrinogen (*p* < 0.001), and shortened activated partial thromboplastin time (APTT; *p* = 0.026) when compared to the control group. (ii) After accounting for potential confounders in linear regression, fibrinogen, and D‐dimer were negatively related to the motor NCV, motor amplitude values, and mean velocity and amplitude. Also, fibrinogen was associated with higher Michigan neuropathy screening instrument (MNSI) scores (*β* 0.140; *p* = 0.001). This result of fibrinogen can be validated in the validation cohort with 317 diabetic patients. (iii) Fibrinogen was independently associated with the risk of DPN (OR 1.172; *p* = 0.035). In the total age group, DPN occurred at a slower rate until the predicted fibrinogen level reached around 3.75 g/L, after which the risk sharply escalated.

**Conclusions:**

Coagulation function is warranted to be concerned in patients with type 2 diabetes to predict and prevent the occurrence of DPN in clinical practice.

## Graphical abstract

This multicenter study, which involved 1211 patients with type 2 diabetes, aimed to elucidate the correlation between coagulation function and the risk and severity of diabetic peripheral neuropathy (DPN). Among the coagulation parameters examined, fibrinogen was found to be independently associated with the risk (OR 1.172; *p* = 0.035) and severity of DPN (*p* < 0.05), and this finding was externally validated.

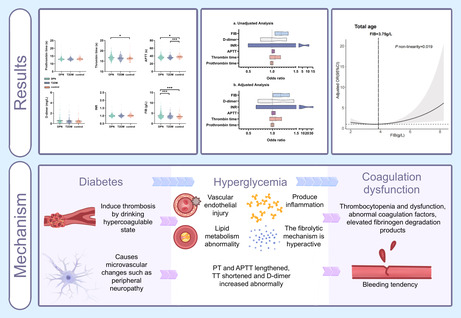

## 1 BACKGROUND

Diabetic peripheral neuropathy (DPN), one of the most common chronic complications of diabetes mellitus,^1^ is estimated to occur in 10%–15% of newly diagnosed diabetics and to reach 50% in diabetic patients.^2^, ^3^ While the 3‐year survival rate for diabetic patients with DPN is approximately 53%.^4^ DPN is a symmetrical length‐dependent sensorimotor polyneuropathy that arises from chronic hyperglycemia, relevant metabolic disorders, cardiovascular risk factors, and microvascular alterations.^5^ The characteristics of DPN, including neuropathic symptoms, paresthesia, and loss of sensation, lead to a rising risk of burns, injuries, and diabetic foot ulcers.^6^, ^7^ Recent research indicated that neuropathy may develop not only during diabetes but also in the prediabetic state.^8^

It is widely recognized that several biomarkers play a role in the diagnosis and prognosis of DPN,^9^ such as age, duration of diabetes, hypertension, dyslipidemia, and smoking.^8^ Individuals with diabetes, encompassing both type 1 and type 2, may experience a hypercoagulable state with elevated clotting factors^10^ and a prethrombotic environment due to prolonged thrombolysis time.^11^ Furthermore, pathological and laboratory studies have demonstrated that even low levels of systemic inflammation may result in the loss of both myelinated and unmyelinated nerve fibers, leading to disruptions in the blood‐nerve barrier and microvascular system,^9^, ^12^ which has been identified as a potential mechanism leading to type 2 diabetes mellitus (T2DM) and its subsequent complications.^13^, ^14^ Nevertheless, the detection of inflammatory markers can be technically challenging and costly, which limits their widespread use in routine clinical practice.

The dysfunction of coagulation function can significantly impact microvascular changes, which has been widely recognized to elevate the risk of DPN in patients with T2DM.^15^, ^16^ Fibrinogen (FIB), the primary protein involved in blood coagulation, was reported to have a strong association with chronic systemic inflammation compared with hypercoagulability.^17^ In a study by Lianlian et al., FIB was identified as an independent risk factor for predicting type 2 diabetic nephropathy,^18^ a key microvascular complication that can lead to kidney failure. Additionally, Zhuang et al. found that T2DM patients with a D‐dimer level of ≥0.22 mg/L had a higher risk of developing DPN.^15^ To date, no research has assessed the impact of coagulation function on the severity of DPN based on electromyography (EMG), nor has any such research been stratified by age groups.

In this retrospective study, we aimed to investigate the potential of coagulation biomarkers in predicting the risk of DPN (the diagnosis based on EMG and neurological assessment) in Chinese patients with T2DM. Our goal was to assess the value of these biomarkers in aiding diagnosis and estimating prognosis in patients across various age groups.

## 2 MATERIALS AND METHODS

### 2.1 Patients

In the main cohort, patients who received nerve conduction examinations were recruited from the First Affiliated Hospital of Wenzhou Medical University between September 2018 and October 2022. Those with type 2 diabetic peripheral neuropathy were categorized into the DPN group (*n* = 614), while those with type 2 diabetes mellitus but without DPN were assigned to the T2DM group (*n* = 280). The remaining individuals without T2DM were selected as the control group (*n* = 133). In the external validation cohort, 196 patients with DPN and 121 patients with T2DM were also enrolled. A total of 317 individuals were included at Zhejiang Provincial People's Hospital, Ruian People's Hospital, Shaoxing People's Hospital in Zhejiang Province, and Anyang District Hospital in Henan Province, from October 2021 to October 2022 (Supplementary Figure S1).

### 2.2 Inclusion and exclusion criteria

The inclusion criteria of the main cohort and external validation were patients with diabetes aged 18 years or older who had undergone nerve conduction examination and Michigan Neuropathy Screening Instrument (MNSI). The exclusion criteria of the main cohort and the external validation gathered: Patients with (1) type 1 diabetes mellitus; special type diabetes mellitus; (2) lack of coagulation function data; (3) severe diseases (liver disease, hematological disorder like hemophilia and venous thromboembolism, kidney disease, malignant disease, respiratory diseases, heart failure, acute infections); (4) stroke; acute cerebral infarction and any neuropathy other than diabetes‐related.^19^ Hence, a total of 1211 patients with diabetes were included in the analysis, comprising 894 from the main cohort at the First Affiliated Hospital of Wenzhou Medical University and 317 from external validation (94 from Zhejiang Provincial People's Hospital, 92 from Ruian People's Hospital, 76 from Shaoxing People's Hospital, and 55 from Anyang District Hospital, respectively) (Supplementary Figure S1).

### 2.3 Clinical assessment

Clinical data were obtained from the patient's electronic history. The data included demographic information: age, course of diabetes, smoking history, hypertension and/or hyperlipidemia history, and BMI. Laboratory data constituted: HbA1c, fasting plasma glucose (FPG), total cholesterol (TC), triglycerides (TG), C‐reaction protein (CRP), and coagulation function indicators such as FIB, prothrombin time (PT), thrombin time (TT) and activated partial thromboplastin time (APTT), international normalized ratio (INR), and D‐dimer. Diabetes complications were also recorded. Results were compared with normal reference ranges. The study protocol was approved by the Ethics Committee of the First Affiliated Hospital of Wenzhou Medical University, Zhejiang Provincial People's Hospital, Ruian People's Hospital, Shaoxing People's Hospital, and Anyang District Hospital, and all participants provided written informed consent to participate in this study.

### 2.4 Neurological symptoms and examinations

The effects of confounding variables were minimized before (e.g., 24 h before) and during neuropathy detection: Stop or avoid drugs (such as beta‐blockers), foods (such as spicy foods), or beverages (such as coffee) that affect heart rate or neurological function; avoid or reduce previous physical activity and emotional stress. The patients with symptomatic hyperglycemia, hypoglycemia, or ketonuria were excluded, or these symptoms were corrected beforehand. All the tests were conducted in a quiet laboratory.^20^

Neurological symptoms of DPN include burning, numbness, tingling, fatigue, cramps, and pain in the leg and/or foot. Neurological signs were defined as reduced or absence of ankle reflexes (using an appropriate tendon reflex hammer) along with reduced or absence of distal sensation, including vibratory sensation (using a standard 128 Hz tuning fork), tactile perception (using a 10 g monofilament on 5 sits per foot), temperature sensation (using cold and warm objects), tingling sensation (using pins), and proprioception.^21^

### 2.5 Nerve conduction examination

The nerve conduction examination was conducted at a room temperature of 24°C, with the legs warmed using an electric heating pad for at least 10 min to achieve a skin temperature of 32–35°C. A skilled technician performed all tests. The nerve conduction velocities (NCV) and nerve conduction amplitudes were measured for both sides of the upper and lower limbs, including motor and sensory branches of the median nerve; motor and sensory branches of the ulnar nerve; motor branch of peroneal nerve; motor branch of the tibial nerve; sensory branch of the superficial peroneal nerve. In addition, both sides of the tibial nerve F‐wave were recorded, and the lower one was regarded as the final F‐wave. Slowed/blocked nerve conduction was defined as more than 2.5 SD below the control nerve conduction threshold. Nerve conduction is defined as abnormal when two or more nerve abnormalities are detected.^22^ DPN was defined as the presence of abnormal nerve conduction together with neurological symptoms or signs.^20^ Vibration‐sensing thresholds of the first metatarsal and tibia (10 cm below the knee) were determined by additional probes (vibrometer).

The velocities and amplitudes of all peripheral nerves examined in the study were standardized, and the standardized values for each patient were aggregated into the corresponding *Z* score. Slow/low nerve conduction (defined as velocity/amplitude *Z* score <−1, and approximately equal to the lowest tertile of the *Z* score) is diagnosed as abnormal nerve conduction by trained electrophysiologists.^23^ The amplitude and velocity *Z* scores for males and females were calculated by the following formulas^24^:

$$\text{Amplitude}:Z_{k_{i}}=\frac{X_{k_{i}}-\bar{X_{k}}}{S_{k}},\text{conduction velocity}:Z_{k_{i}}=\frac{X_{k_{i}}-\bar{X_{k}}}{S_{k.}}$$

$$\text{Summed amplitude}:Z_{i}=\sum Z_{k_{i}}$$

$$\text{Summed velocity}:Z_{i}=\sum Z_{k_{i}}$$

$$k=\left(\text{right motor ulnar},\text{left motor ulnar}\ldots \mathrm{two}\;\text{sides of each nerve}\right)$$

i=1,2,3,…n

### 2.6 Michigan neuropathy screening instrument

The MNSI score is a comprehensive tool that includes both a 15‐item self‐administered questionnaire and a structured examination of the feet (MNSIE). The MNSIE is scored for abnormalities of appearance, presence of ulcers, vibration perception at the distal great toe, and ankle reflexes. The score for each parameter is as follows: appearance of feet (normal = 0, abnormal = 1), ulceration (absent = 0, present = 1), ankle reflexes (absent = 1, present with reinforcement = 0.5, present = 0), and vibration perception (absent = 1, reduced = 0.5, present = 0). A trained and certified health professional performs the MNSIE to reduce interobserver variability. Evaluation of each parameter is made on both sides, and the maximum score is 8 points.^25^ A higher score indicates more severe neuropathy in the patient.

### 2.7 Definition of disease

T2DM was diagnosed based on blood glucose measurements, medication data (linked to pharmacy dispensing data), and records from general practitioners. Patients with fasting glucose ≥7.0 mmol/L or nonfasting glucose levels ≥11.1 mmol/L (if there is no fasting glucose sample available), and those receiving glucose‐lowering therapy were considered to have T2DM.^26^

Abnormal nerve conduction is defined as the abnormality in two or more measured nerves. The presence of nerve conduction abnormality along with one or more neurological symptoms or signs is confirmed DPN.^20^ When any of the three elements of the screening process—symptoms, signs, or abnormal nerve conduction parameters—was abnormal, medical records would be scrutinized to see if these participants had accepted a previous DPN diagnosis by a specialist. Participants were allowed to self‐report any previous diagnosis of DPN, which was later verified and recorded. All data obtained from each participant's screening process and medical records underwent evaluation by a panel of experts with considerable experience in diagnosing neuromuscular diseases. This panel included a seasoned neuromuscular specialist, a neurophysiologist, and a physician with expertise in epidemiology.

### 2.8 Statistical analysis

IBM SPSS version 25.0 software (IBM Corp., Armonk, NY, USA) was used to perform all analyses. For continuous variables, the unpaired Student's *t*‐test was adopted for those with normal distribution, the Wilcoxon rank sum test or Kruskal–Wallis test was applied for abnormal distribution, and the Chi‐square test or Fisher's exact test was used for the dichotomous variables. Normally distributed continuous variables were expressed as the mean ± standard error of the mean (mean ± SEM), while nonnormally distributed continuous variables were expressed as median and quartile ranges (median, IQR). The dichotomous variables were presented as percentages (%). Spearman's test was used to explore the correlation between FIB and EMG parameters. After adjusting for age, course of diabetes, hypertension, hyperlipidemia, BMI, smoking, FPG, HbA1c, TG, TC, and CRP, multiple linear regression analysis was carried out to further analyze the relationship between the levels of coagulation function, abnormal nerve conduction, and MNSI exam. Multivariate logistic regression analysis was performed to determine the relationship between coagulation function and the risk of DPN, manifesting that serum FIB served as an independent factor for the occurrence of DPN. Restricted cubic spline curves with three knots (defined at the 10th, 50th, and 90th percentiles) for the development of DPN were further plotted to provide more precise estimates and examine the nonlinear relationships. All tests were bilateral, and *p* < 0.05 was considered statistically significant.

## 3 RESULTS

### 3.1 The distribution of coagulation function indicators in three groups

**FIGURE 1:**
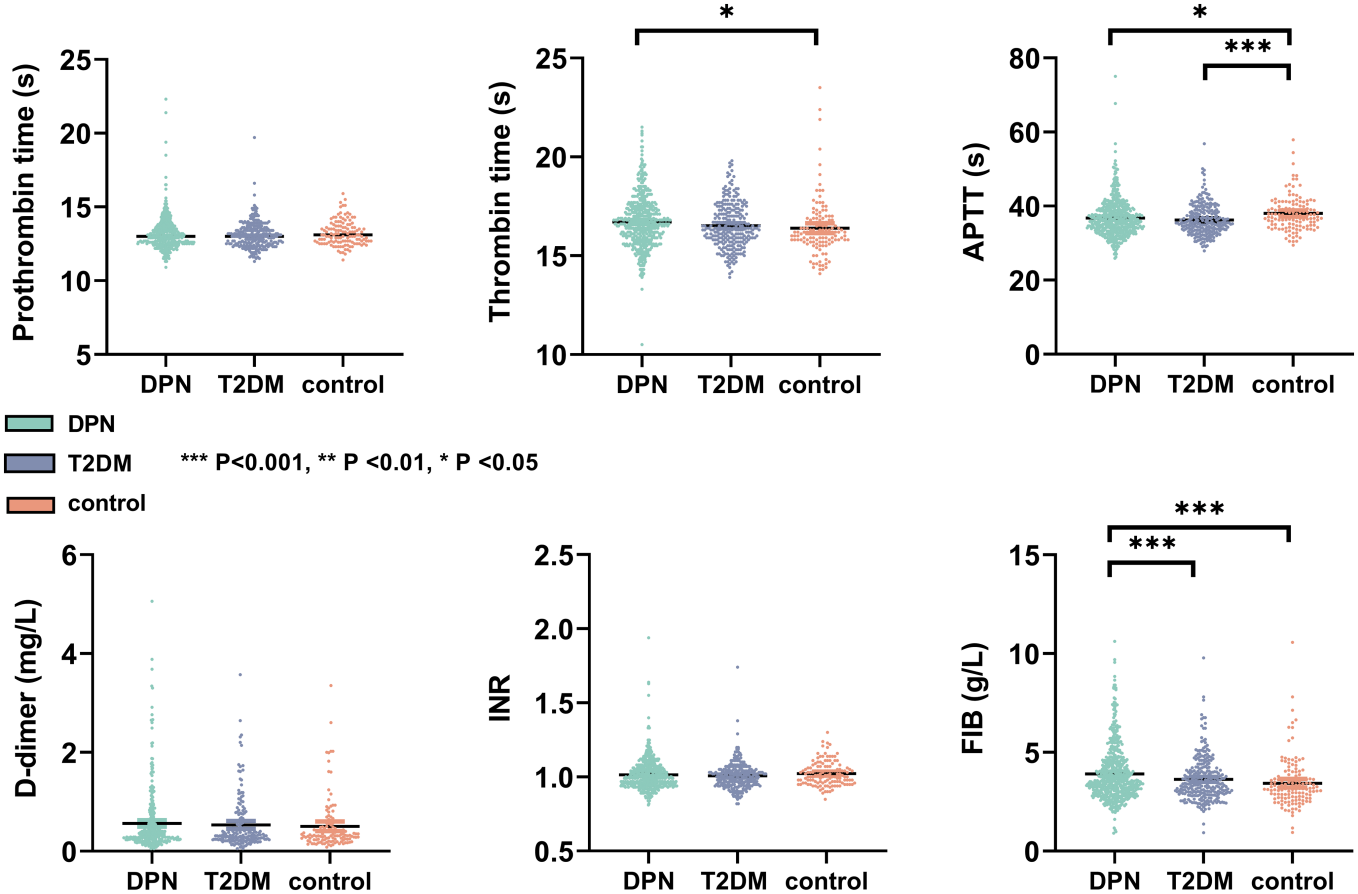
Distribution of coagulation function parameters in each disease group. The distribution of coagulation function parameters (Prothrombin time, thrombin time, APTT, INR, D‐dimer, and FIB) in DPN, T2DM, and control groups. DPN group patients with type 2 diabetic peripheral neuropathy, T2DM group patients with type 2 diabetes without DPN, control group individuals without diabetes, APTT, activated partial thromboplastin time; FIB, fibrinogen; INR, international normalized ratio. ***p < 0.001, **p < 0.01, *p < 0.05.

In the main cohort, after applying the inclusion and exclusion criteria, a total of 614 patients diagnosed with type 2 diabetic peripheral neuropathy (DPN group), 280 patients with type 2 diabetes without DPN (T2DM group), and 133 individuals without diabetes (control group) were recruited from the First Affiliated Hospital of Wenzhou Medical University and underwent EMG examination. The distribution of coagulation function indicators among the DPN, T2DM, and control groups is illustrated in Figure 1. Compared to the control group, the DPN group showed longer TT (*p* = 0.012) and shorter APTT (*p* = 0.026), while exhibiting higher levels of FIB (*p* < 0.001). Notably, the DPN group demonstrated significantly higher levels of FIB (*p* < 0.001) than the T2DM group. Conversely, there were no significant differences in PT, INR, and D‐dimer levels among the three groups.

### 3.2 Coagulation function and EMG parameters

In the main cohort, the potential correlation between coagulation function and EMG parameters was assessed using multiple linear regression (Table 1 **)**. Notably, FIB levels exhibited a negative association with the NCV and amplitude of both upper and lower limbs, except superficial peroneal sensory amplitude, after adjusting for possible confounding factors (all *p* < 0.03). Furthermore, FIB levels were found to be significantly correlated with reduced mean motor nerve amplitude (MNAmp; *β* = −0.464, *p* < 0.001), mean motor nerve conduction velocity (MNCV; *β* = −0.644, *p* < 0.001), mean sensory nerve amplitude (SNAmp; *β* = −1.595, *p* < 0.001), and mean sensory nerve conduction velocity (SNCV; *β* = −0.589, *p* < 0.001). Moreover, D‐dimer levels were observed to be associated with reduced motor NCV and motor amplitude (*p* < 0.01) and were significantly correlated with depressed MNAmp (*β* = −1.224, *p* < 0.001), MNCV (*β* = −1.251, *p* < 0.001), whereas no association with EMG parameters was identified for other coagulation function indicators, such as PT, TT, APTT, and INR.

**TABLE 1:** Multiple linear regression analysis of the correlation between coagulation function and electromyography parameters (*N* = 894).

Electromyography parameters	PT		TT		APTT		INR		D‐dimer		FIB	
	*β*	*p* Value	*β*	*p* Value	*β*	*p* Value	*β*	*p* Value	*β*	*p* Value	*β*	*p* Value
Motor NCV (m/s)												
Ulnar	0.042	0.355	−0.083	0.576	−0.030	0.494	−0.488	0.846	−1.798	<0.001	−0.798	<0.001
Median	−0.007	0.869	−0.098	0.464	−0.016	0.675	−0.743	0.742	−1.244	0.004	−0.783	<0.001
Peroneal	0.008	0.828	−0.328	0.006	−0.056	0.110	−2.889	0.143	−1.115	0.006	−0.534	<0.001
Tibial	−0.022	0.548	−0.371	0.002	−0.045	0.194	−2.913	0.131	−1.235	<0.001	−0.487	<0.001
Sensory NCV (m/s)												
Ulnar	0.045	0.313	−0.012	0.933	−0.046	0.280	−4.183	0.088	−0.619	0.189	−0.534	0.004
Median	−0.145	0.012	0.068	0.718	−0.021	0.705	−5.174	0.105	−0.779	0.181	−0.540	0.022
Sup peroneal	0.026	0.532	0.036	0.799	0.031	0.479	1.495	0.510	0.736	0.154	−0.424	<0.001
Motor amplitude values												
Ulnar	−0.026	0.321	0.154	0.072	−0.009	0.724	0.306	0.832	−1.231	<0.001	−0.338	0.002
Median	−0.033	0.163	0.021	0.785	−0.059	0.009	−1.133	0.389	−1.070	<0.001	−0.290	0.003
Peroneal	0.009	0.740	−0.09	0.312	−0.018	0.486	1.042	0.475	−1.009	<0.001	−0.487	<0.001
Tibial	−0.124	0.007	−0.315	0.035	−0.107	0.012	−2.843	0.240	−1.548	<0.001	−0.802	<0.001
Sensory amplitude values												
Ulnar	0.196	0.199	−0.748	0.135	−0.139	0.342	−7.016	0.405	−2.262	0.171	−1.878	0.003
Median	−0.035	0.812	−1.422	0.003	0.073	0.607	−6.959	0.395	−2.493	0.118	−1.692	0.005
Sup peroneal	0.033	0.662	−0.051	0.843	−0.086	0.279	−1.973	0.673	0.872	0.390	−0.441	0.211
F‐wave minimum latency (ms)	−0.016	0.659	0.422	<0.001	0.068	0.050	1.168	0.550	1.167	0.002	0.558	<0.001
Mean velocity/amplitude												
MNAmp	−0.040	0.091	−0.087	0.247	−0.043	0.043	0.227	0.854	−1.224	<0.001	−0.464	<0.001
MNCV	0.009	0.789	−0.255	0.018	−0.035	0.263	−1.719	0.330	−1.251	<0.001	−0.644	<0.001
SNAmp	0.078	0.472	−0.822	0.018	−0.014	0.893	−2.869	0.622	−2.089	0.059	−1.595	<0.001
SNCV	−0.020	0.610	0.047	0.708	−0.011	0.772	−2.638	0.196	−0.526	0.177	−0.589	<0.001
Summed *Z* scores												
Amplitude *Z* score	−0.027	0.352	−0.105	0.303	−0.044	0.162	−1.432	0.404	−0.392	0.339	−0.240	0.100
Velocity *Z* score	−0.007	0.823	−0.117	0.307	−0.036	0.305	−3.021	0.117	0.202	0.628	−0.228	0.169

*Note*: Adjusted for age, course of diabetes, hypertension, hyperlipidemia, BMI, smoking, FPG, HbA1c, TG, TC and CRP.

Abbreviations: APTT, activated partial thromboplastin time; FIB, fibrinogen; INR, international normalized ratio; MNAmp, mean motor nerve amplitude; MNCV, mean motor nerve conduction velocity; NCV, nerve conduction velocity; PT, prothrombin time; SNAmp, mean sensory nerve amplitude; SNCV, mean sensory nerve conduction velocity; TT, thrombin time.

In the external validation cohort, the baseline characteristics of 196 patients with DPN and 121 patients with type 2 diabetes without DPN are detailed in Supplementary Table S1. The DPN group exhibited higher MNSI scores (*p* < 0.001) and FIB levels (*p* = 0.021) compared to the T2DM group. In multiple linear regression, we found that FIB levels were negatively associated with motor amplitude values (*p* < 0.01) and significantly correlated with the reduced MNAmp (*β* = −0.793, *p* < 0.001), MNCV (*β* = −0.830, *p* < 0.001), SNAmp (*β* = −2.664, *p* < 0.001), and SNCV (*β* = −0.783, *p* < 0.001, Supplementary Table S2).

### 3.3 Coagulation function and MNSI exam

In the main cohort, through multivariate linear regression analysis, we discovered that FIB levels were associated with higher MNSI scores after adjusting for various DPN‐related indicators, indicating severe neuropathy (*β* = 0.140, 95% CI: 0.056, 0.224, *p* = 0.001), whereas no significant association was observed with other coagulation biomarkers (Table 2). In the external validation, the relationship between FIB and MNSI exam in multivariate linear regression analysis was evident, after adjusting for various DPN‐related indicators (*β* = 0.223, 95% CI: 0.027–0.419, *p* = 0.026, Table 2). TT levels were negatively associated with the severity of neuropathy (*β* = −0.165, 95% CI: −0.283, −0.048, *p* = 0.006), whereas no significant association was observed between other coagulation biomarkers and MNSI exam. Importantly, the results of multiple linear regression validated the finding that FIB levels were positively correlated with the severity of neuropathy in both the main cohort and the validation cohort, consistent with the outcomes presented in Table 1.

**TABLE 2:** Multiple linear regression analysis of the correlation between coagulation function and MNSI exam in both the main cohort and external validation.

Coagulation function	*β*	95% CI	*p* Value
Main cohort (*n* = 894)			
Prothrombin time	0.001	−0.020, 0.021	0.957
Thrombin time	−0.013	−0.081, 0.055	0.710
APTT	−0.006	−0.026, 0.014	0.526
INR	−0.157	−1.250, 0.931	0.776
D‐dimer	0.121	−0.111,0.353	0.304
FIB	0.140	0.056, 0.224	0.001
External validation (*n* = 317)			
Prothrombin time	−0.022	−0.149, 0.105	0.732
Thrombin time	−0.165	−0.283, −0.048	0.006
APTT	0.048	−0.011, 0.104	0.113
INR	0.730	−0.429,1.890	0.215
D‐dimer	−0.066	−0.257, 0.126	0.497
FIB	0.223	0.027, 0.419	0.026

*Note*: Adjusted for age, course of diabetes, hypertension, hyperlipidemia, smoking, FPG, HbA1c, TG, TC, and CRP.

Abbreviations: APTT, activated partial thromboplastin time; FIB, fibrinogen; INR, international normalized ratio.

### 3.4 Coagulation function and diabetic peripheral neuropathy

**FIGURE 2:**
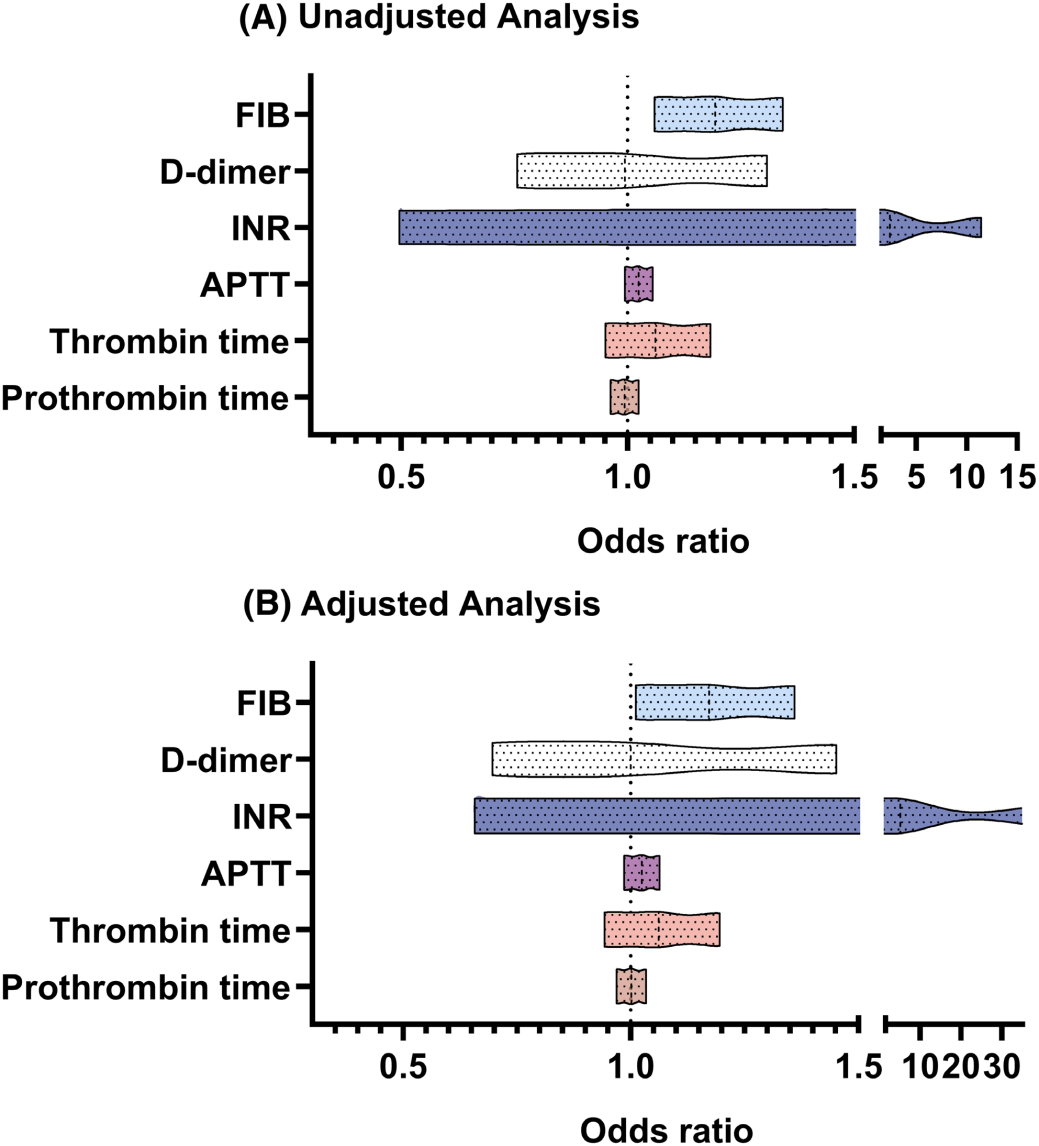
The odds ratio (95% confidence interval) of the presence of DPN in unadjusted analysis (A) and adjusted analysis (B). APTT, activated partial thromboplastin time; FIB, fibrinogen; INR, international normalized ratio.

In the main cohort, the relationship between coagulation function and the risk of DPN in type 2 diabetic patients in logistics regression is shown in Figure 2. After adjusting for potential confounders, serum FIB was found to be independently correlated with the incremental odds of DPN in the multivariate logistic analysis (adjusted OR, 1.172 (1.011–1.360), *p* = 0.034). However, concerning other coagulation function biomarkers, like PT, TT, APTT, INR, and D‐dimer, the results were insignificant. Given that FIB emerged as an independent predictor of the occurrence and severity of DPN, a more detailed analysis using EMG was conducted in the study.

### 3.5 Unstandardized parameters and standardized *Z* score of EMG by tertiles of fibrinogen

Further analysis of EMG parameters after tripartite grouping according to FIB levels in the main cohort with 894 patients is performed in Table 3. The age and proportion of patients with DPN increased with higher FIB levels. The motor and sensory nerve conduction parameters of upper and lower limbs including NCV, amplitude, summed amplitude *Z* score (1.91 ± 0.26 vs. 0.99 ± 0.25 and −0.17 ± 0.28, *p* < 0.001), and summed velocity *Z* score (1.52 ± 0.27 vs. 1.54 ± 0.30 and 0.01 ± 0.30, *p* < 0.001) of low FIB group (T1, <3.11 g/L) were significantly higher than the middle group (T2, 3.11–3.96 g/L) and the high group (T3, >3.96 g/L).

**TABLE 3:** The presence of DPN and parameters of electromyography by tertiles (T1–T3) of FIB (*N* = 894).

Patients	FIB			
	T1	T2	T3	*p* Value
	<3.11	3.11–3.96	>3.96	
Number	281	303	310	
Age	54.87 ± 0.67	58.94 ± 0.64	61.88 ± 0.61	<0.001
FIB	2.64 ± 0.02	3.48 ± 0.01	5.19 ± 0.07	<0.001
DPN%	180 (52.9%)	206 (59.4%)	228 (67.1%)	0.002
Motor NCV (m/s)				
Ulnar	51.95 ± 0.32	52.23 ± 0.38	49.58 ± 0.38	<0.001
Median	53.08 ± 0.28	52.74 ± 0.32	50.77 ± 0.31	<0.001
Peroneal	43.98 ± 0.29	43.95 ± 0.29	41.92 ± 0.29	<0.001
Tibial	44.55 ± 0.30	44.36 ± 0.27	42.48 ± 0.28	<0.001
Sensory NCV (m/s)				
Ulnar	52.27 ± 0.34	52.92 ± 0.35	50.82 ± 0.37	<0.001
Median	52.65 ± 0.41	51.99 ± 0.43	49.42 ± 0.45	<0.001
Sup Peroneal	45.81 ± 0.30	45.03 ± 0.32	44.11 ± 0.32	<0.001
Motor amplitude values				
Ulnar	12.98 ± 0.20	12.20 ± 0.17	11.34 ± 0.19	<0.001
Median	12.85 ± 0.18	12.43 ± 0.21	10.98 ± 0.18	<0.001
Peroneal	7.69 ± 0.26	6.53 ± 0.20	5.26 ± 0.19	<0.001
Tibial	14.88 ± 0.33	13.24 ± 0.34	10.99 ± 0.33	<0.001
Sensory amplitude values				
Ulnar	36.79 ± 1.18	36.20 ± 1.14	30.44 ± 1.30	<0.001
Median	38.23 ± 1.25	37.41 ± 1.13	30.10 ± 1.17	<0.001
Sup peroneal	14.02 ± 0.63	12.84 ± 0.61	11.14 ± 0.43	0.003
F‐wave minimum latency(ms)	44.59 ± 0.25	44.64 ± 0.27	46.53 ± 0.29	<0.001
Mean velocity/amplitude				
MNAmp	12.11 ± 0.21	10.94 ± 0.17	9.52 ± 0.18	<0.001
MNCV	48.06 ± 0.26	47.79 ± 0.27	45.89 ± 0.26	<0.001
SNAmp	28.45 ± 0.83	27.33 ± 0.78	22.44 ± 0.78	<0.001
SNCV	49.97 ± 0.27	49.45 ± 0.29	47.75 ± 0.29	<0.001
Summed *Z* scores				
Amplitude *Z* score	1.91 ± 0.26	0.99 ± 0.25	−0.17 ± 0.28	<0.001
Velocity *Z* score	1.52 ± 0.27	1.54 ± 0.30	0.01 ± 0.30	<0.001

*Note*: Data are means ± SEM or *n* (%).

Abbreviations: DPN, diabetic peripheral neuropathy group; FIB, plasma fibrinogen; MNAmp, motor nerve amplitude; MNCV, motor nerve conduction velocity; NCV, nerve conduction velocity; SNAmp, sensory nerve amplitude, SNCV, sensory nerve conduction velocity; T1, tertile 1; T2, tertile 2; T3, tertile 3.

Stratification according to three age groups (<55 years, 55–65 years, and >65 years) revealed that the differences in motor NCV, motor amplitude values, MNAmp, MNCV, SNAmp, and SNCV among the low, medium, and high FIB groups were still apparent in the group aged under 55 years, the detailed results are listed in Supplementary Table S3. Similar results were also observed in the groups aged more than 55 years.

### 3.6 Serum fibrinogen and diabetic peripheral neuropathy stratified by age

In the main cohort, correlation analysis with Spearman's test revealed a negative association between FIB levels and NCV and amplitude of upper and lower limbs, with all variables showing a *p* value of <0.001, except for superficial peroneal amplitude and velocity (*p* < 0.01) (Supplementary Table S4). Still, after stratified by age, FIB was correlated with a significant reduction in motor NCV, F‐wave minimum latency, MNAmp, MNCV, SNAmp, and SNCV in each age group (*p* < 0.03).

**FIGURE 3:**
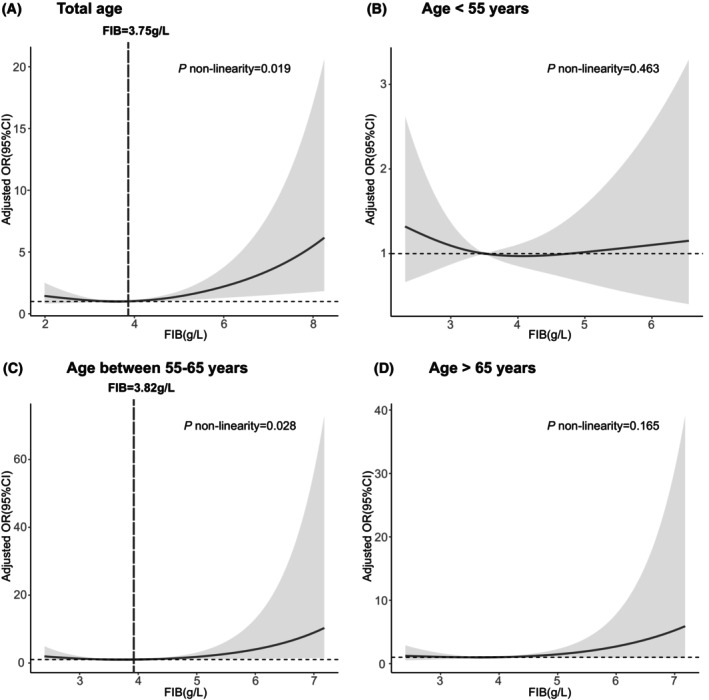
Multivariable adjusted odd ratios for DPN according to levels of FIB on (A) all groups, (B) group aged <55 years, (C) group aged between 55 and 65 years, and (D) group aged >65 years. Multiple spline regression analysis with three knots is utilized in DPN. The solid line indicates the odds ratio, while the shadow indicates 95% CI. Reference lines for no association are indicated by the dashed lines at an odd ratio of 1.0. Data are adjusted for age, course of diabetes, hypertension, hyperlipidemia, BMI, smoking, FIB, FPG, HbA1c, TG, TC, and CRP. DPN, diabetic peripheral neuropathy; FIB, plasma fibrinogen; FPG, fasting plasma glucose; TC, total cholesterol; TG, triglycerides.

Also, the nonlinear relationship between elevated FIB and the higher risk of DPN was further manifested in the main cohort with restricted cubic spline regressions with three knots (Figure 3). In the total age group, the development of DPN decreased until the predicted FIB levels reached 3.75 g/L, and then increased thereafter (*p* for nonlinearity = 0.019). A similar trend was observed in the group aged between 55 and 65 years, where the risk of DPN was relatively stable until around 3.82 g/L of the predicted FIB levels and then increased thereafter (*p* for nonlinearity = 0.028). However, no nonlinear relationship was found between FIB levels and DPN in groups with ages <55 years or >65 years. In the validation cohort, the risk of developing DPN decreased until the predicted FIB level reached 3.78 g/L, after which it increased sharply (Supplementary Figure S2; *p* nonlinearity = 0.027).

## 4 DISCUSSION

In light of our findings, we systemically and comprehensively examined the relationship among coagulation function, the prevalence of DPN, and clinical nerve conduction characteristics in 1211 individuals from five hospitals, including the First Affiliated Hospital of Wenzhou Medical University, Zhejiang Provincial People's Hospital, Ruian People's Hospital, Shaoxing People's Hospital, and Anyang District Hospital. Our results indicated that FIB and D‐dimer were negatively associated with EMG parameters in multiple linear regression models, after adjusting for potential confounders. Also, FIB levels were positively associated with higher MNSI scores. This result of FIB can be validated in the external cohort of 317 diabetic patients. Among coagulation function indicators, FIB was an independent predictor of DPN in patients with T2DM and positively correlated with the severity of this disease (described by the decreased value of NCV and amplitude and higher MNSI scores). Furthermore, even after stratifying by age, the severity of DPN continued to increase with rising FIB levels. Additionally, the nonlinear relationship between FIB levels and DPN was observed.

**FIGURE 4:**
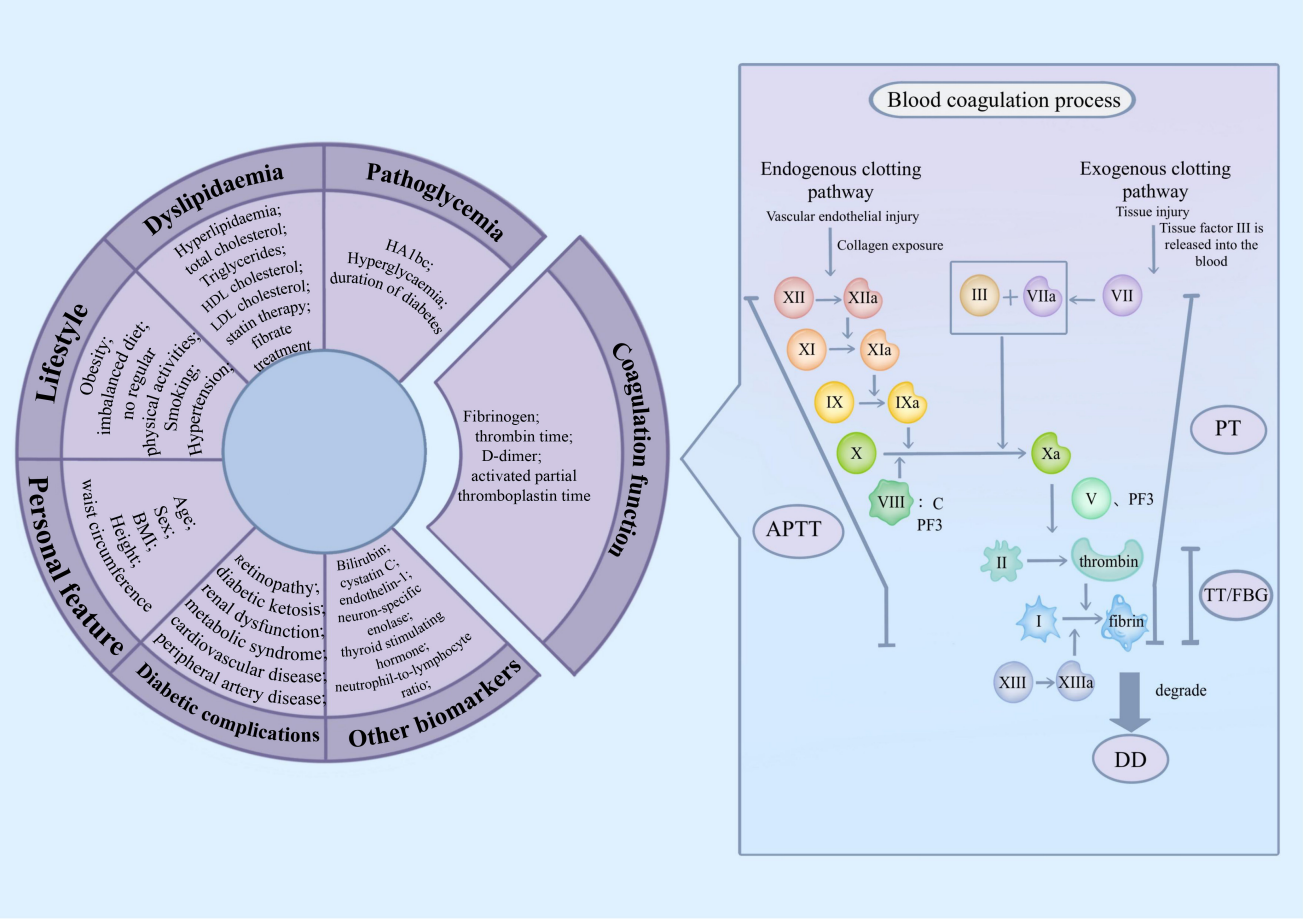
Schematic diagram of multiple influences affecting DPN. The left panel represents the biomarkers associated with DPN and the right panel represents the coagulation pathways involved in the coagulation index.

Coagulation function dysfunction plays a crucial role in the occurrence and development of diabetic microvascular complications. Diabetes‐induced thrombotic propensity and hypercoagulability,^11^ along with chronic systemic inflammation^9^, ^12^ have all been identified as potential mechanisms underlying neuropathy (Figure 4). PT and APTT reflect the exogenous and endogenous functions of the thrombin system, respectively. TT reflects the conversion time of fibrinogen to fibrin, and D‐dimer is a degradation product of fibrinogen.^15^ Elevated levels of hyperlipemia and hyperglycemia induce chronic systemic inflammation, which can lead to platelet activation and disorder of the coagulation and anticoagulation systems, ultimately resulting in hypercoagulability and thrombosis.^27^ This exacerbates the course of diabetes and increases the risk of complications, such as the occurrence of DPN.^28^ FIB, a major component of the temporary extracellular matrix formed after tissue damage, participates in blood clotting and is associated with insulin resistance syndrome and thrombosis, thereby promoting a state of hypercoagulation and hyperviscosity in the blood.^29^ Moreover, FIB can directly participate in coagulation and combine with platelet membrane protein IIb/IIIa to mediate platelet aggregation and ultimately lead to widespread microthrombosis, resulting in severe microvascular disease.^30^

Pan et al. screened several clinical frequently‐used coagulation indicators and then concluded that FIB was an independent risk factor for predicting type 2 diabetic nephropathy, and the main microvascular complication leading to kidney failure.^18^ In a single‐center study with a total of 561 subjects, the *k* value and angle *α*, reflecting the plasma FIB function, were discovered to be associated with the early diagnosis of DPN. They revealed that compared with diabetic patients without DPN, the level of FIB and the angle α were higher, and the *k* value was lower in patients with DPN.^16^ In contrast to the above study, our study paid attention to the nerve conduction obtained from EMG, applied a large sample of nerve conduction studies, and stratified patients based on different ages, to seek a further correlation between FIB and DPN. Moreover, the nerve conduction test, which is the gold standard for diagnosis,^1^ is also employed for diagnosing and measuring the severity of DPN in our study, rather than merely applying questionnaires and physical examinations.

The course of diabetes plays an important role in the development of DPN, as a longer disease course is a potential risk factor for microvascular disease.^28^ Compared to patients with diabetes for <5 years, those with a duration of >5 years were four times more likely to develop microvascular complications.^31^ A cross‐sectional study from Saudi Arabia involving 430 diabetic patients found that those with type 2 diabetes for >10 years were more likely to develop chronic diabetic complications.^32^ Consistent with the previous study, in our study, the course of diabetes was strongly associated with the risk of DPN (adjusted OR, 1.110(1.077–1.146), *p* < 0.001). Prolonged hyperglycemia can damage blood vessels, leading to cellular damage in neurons and Schwann cells of peripheral nerves, which may result in DPN.^33^

The middle‐aged group (aged 55–65 years) is a unique middle‐more time window, during which age‐related chronic diseases may be in the preclinical stage, and symptoms and signs are too mild to be detected by clinicians.^34^ In our study, a significant correlation between high levels of FIB and abnormal nerve conduction was observed at all stages, whereas, there exists a big gap in each age group. For instance, the absolute value of the Spearman correlation coefficient in the middle‐aged group (55 ≤ age ≤ 65) and elder (age > 65) group was much greater than in the younger group (age < 55) for most of the EMG parameters. Additionally, the probability of developing DPN was found to be higher in the middle‐aged group (65.8%) and elder group (67.0%) than in the younger group (52.3%). Hence, in clinical practice, more frequent DPN screening tests or earlier EMG tests for middle‐aged and elder patients with high FIB might increase the proportion of early prevention and treatment and ultimately prevent serious clinical outcomes.

Undoubtedly, several limitations of our study require careful consideration. First, the cross‐sectional study could not eliminate the bias from patients enrolled in the study, as the included patients without the diagnosis of DPN who received nerve conduction examination were more likely to be in the preclinical stage of DPN, thus the predictive value of FIB might be underestimated. However, the large sample size and the use of unified assessment approaches across five centers enhance the reliability of the results. Second, patients with type 1 diabetes mellitus were not included, so the results may not be generalizable. Third, coagulation function‐related biomarkers were determined by one sampling. However, these biomarkers can fluctuate during the course of T2DM and may be affected by antidiabetic medications. Fourth, we included CRP as a covariate in the model, but some inflammatory biomarkers, such as anti‐nuclear antibodies and erythrocyte sedimentation rate, were unavailable in our study. Hence, in future research, data on these inflammatory indicators should be collected prospectively. It is worth mentioning that the integrated application of nerve conduction examination and clinical examination brings about more accuracy and credibility. Additionally, a detailed assessment of nervous function may provide numerous variables, increasing the chances of identifying early risks. By combining the normal deviations of several parameters into an average *Z* score, the number of variables was greatly reduced and the sensitivity of the test could be improved as well.^23^ In the future, further fundamental research is needed to explore the specific pathophysiological mechanism of FIB within T2DM and DPN, and large‐scale prospective studies were expected to obtain a more precise risk threshold of FIB.

In total, coagulation function factors, especially elevated levels of FIB, may be implicated in the development and severity of DPN among Chinese patients with T2DM. Hence, the monitoring of coagulation function factors in patients diagnosed with T2DM could offer valuable insights into potential therapeutic targets and facilitate early intervention to prevent the onset and progression of DPN.

## AUTHOR CONTRIBUTIONS

Jiali Xie, Xinyue Yu, and Luowei Chen contributed to the data statistics and writing the paper. Yifan Cheng, Kezheng Li, Mengwan Song, Fei Feng, and Yunlei Cai were responsible for data acquisition from four subcenters. Yinuo Chen oversaw the production of select figures. Shuting Tong, Yuqin Qian, Yiting Xu, Haiqin Zhang, Junjie Yang, Zirui Xu, Can Cui, and Huan Yu were responsible for the diligent data collection efforts. Finally, Binbin Deng provided crucial resources and designed the study with meticulous attention to detail.

## FUNDING INFORMATION

This work was supported by the National Natural Science Foundation of China (81901273) and the Science Technology Department of Zhejiang Province (Grant no. Q21H090076).

## CONFLICT OF INTEREST STATEMENT

The authors have stated explicitly that there are no conflicts of interest in connection with this article.

## Supporting information


Data S1.

## Data Availability

Data and material can be shared with the consent of the corresponding authors.
