# Supplementary material for: Whether coagulation dysfunction influences the onset and progression of diabetic peripheral neuropathy: A multicenter study in middle‐aged and aged patients with type 2 diabetes
Source: CNS Neurosci Ther. 2024 Sep 11;30(9):e70040. doi: 10.1111/cns.70040 (PMC11388410; doi:10.1111/cns.70040)
Supplement: Supplementary file 1 — Data S1. [file CNS-30-e70040-s001.docx]

**Supplementary Information**

**Whether coagulation dysfunction influence the onset and progression of diabetic peripheral neuropathy: a multi-center study in middle-aged and aged patients with type 2 diabetes**

Jiali Xie^1,2^*, Xinyue Yu^3^*, Luowei Chen^1,4^*, Yifan Cheng^5^, Kezheng Li^1^, Mengwan Song^6,7^, Yinuo Chen^1,6^, Fei Feng^6,8^, Yunlei Cai^9^, Shuting Tong^1^, Yuqin Qian^1,10^, Yiting Xu^1^, Haiqin Zhang^1,6,^ Junjie Yang^1,6,^ Zirui Xu^1,6^, Can Cui^11^, Huan Yu^12^, Binbin Deng^1^

1. Department of Neurology, First Affiliated Hospital of Wenzhou Medical University, Wenzhou, PR. China.

2. Department of Neurology, Shanghai East Hospital, Tongji University School of Medicine, Shanghai, PR. China.

3. Alberta Institute, Wenzhou Medical University, Wenzhou, PR. China.

4. Department of Neurology, The Second Affiliated Hospital of Zhejiang University, School of Medicine, Hangzhou, China.

5. Center for Rehabilitation Medicine, Department of Neurology, Zhejiang Provincial People’s Hospital, Affiliated People’s Hospital, Hangzhou Medical College, 310014 Hangzhou, China

6. First Clinical College of Wenzhou Medical University, Wenzhou, PR. China.

7. Department of Neurology, Ruian People’ s Hospital, Wenzhou, PR, China.

8. Department of Neurology, Shaoxing People’ s Hospital, Shaoxing, PR, China.

9. Anyang District Hospital, Dengta Road, Beiguan District, Anyang City, Henan Province.

10. Department of Neurology, Institute of Neurology, Ruijin Hospital, Shanghai Jiao Tong University School of Medicine, Shanghai, China.

11. Institute of Environmental Medicine, Karolinska Institutet, Stockholm, Sweden

12. Department of Pediatrics, Second Affiliated Hospital and Yuying Children's Hospital of Wenzhou Medical University, Wenzhou, PR. China.

*These authors contributed equally to this work

**Corresponding author:** Binbin Deng, First Affiliated Hospital of Wenzhou Medical University, Wenzhou, PR. China. Telephone:13157150018

Email: dbinbin@aliyun.com

**Supplementary Fig 1- Study recruitment flow chart.**
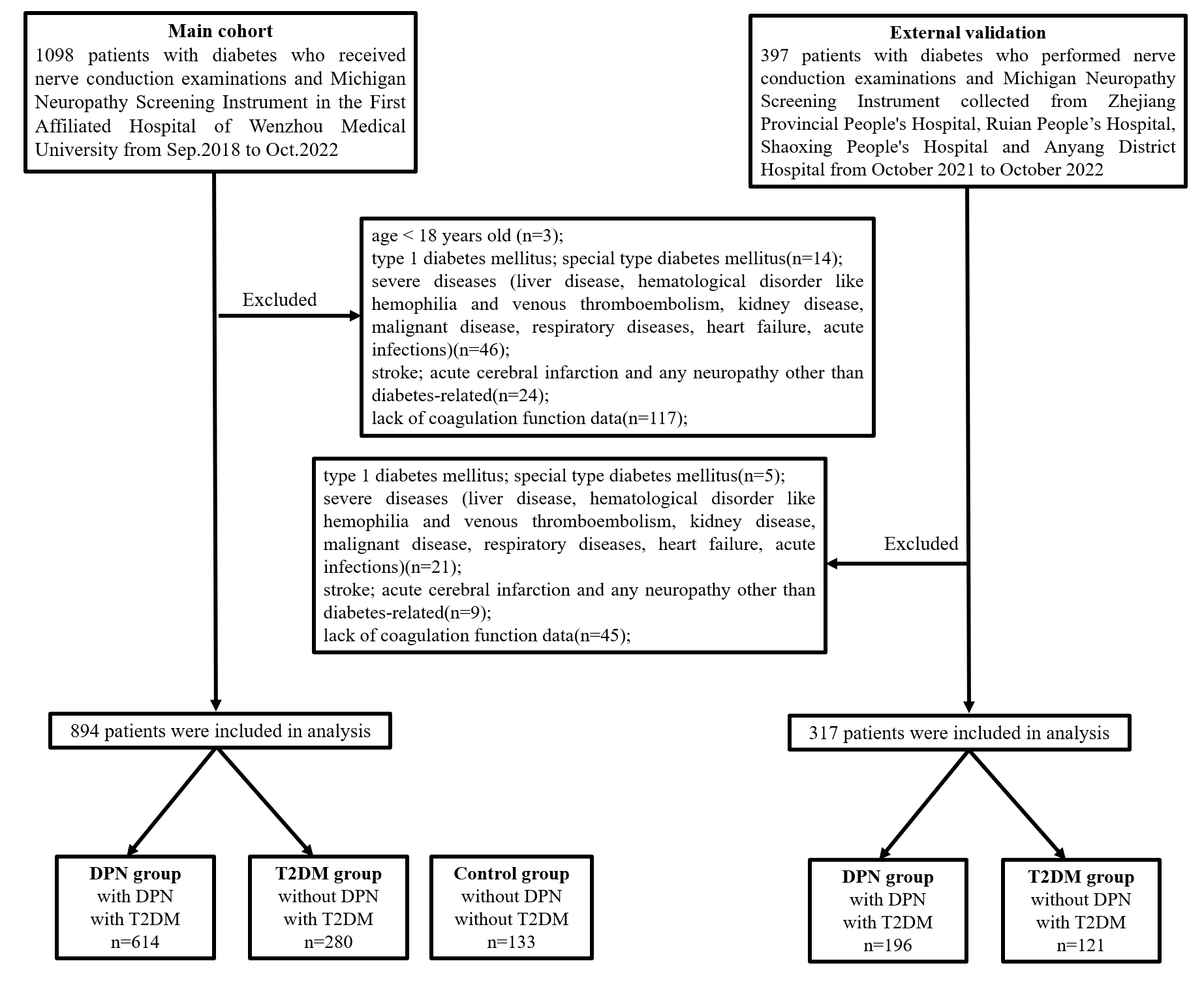


**Supplementary Fig 2- Multivariable adjusted odd ratios for DPN according to levels of FIB in the external validation.**


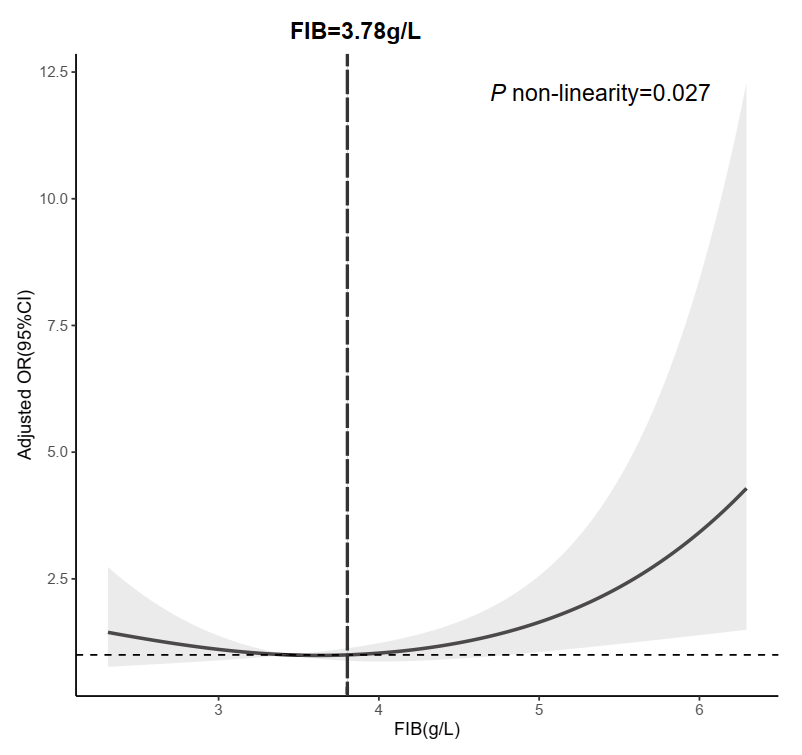


Multiple spline regression analysis with three knots is utilized in DPN. The solid line indicates the odds ratio, while the shadow indicates 95% CI. Reference lines for no association are indicated by the dashed lines at an odd ratio of 1.0. Data are adjusted for age, course of diabetes, hypertension, hyperlipidemia, BMI, smoking, FIB, FPG, HbA1c, TG, TC, and CRP. DPN diabetic peripheral neuropathy, FIB plasma fibrinogen, FPG fasting plasma glucose, TG triglycerides and TC total cholesterol.

| **Supplementary Table 1 –Baseline characteristics of 317 patients enrolled in the multi-center study in the external validation.** | | | |
| --- | --- | --- | --- |
| **Clinical characteristics** | **DPN** | **T2DM** | ***P* value** |
|  | **n=196** | **n=121** |  |
| **Demographic characteristics** |  |  |  |
| Gender(male%) | 115(59.9%) | 66(55.5%) | 0.514 |
| Age (year) | 58.35±1.02 | 58.02±1.43 | 0.844 |
| Duration (year) | 8.61±0.60 | 7.65±0.78 | 0.329 |
| Smoke (%) | 53(27.0%) | 38(31.4%) | 0.480 |
| Hypertension (%) | 105(53.6%) | 55(45.5%) | 0.198 |
| Hyperlipidemia (%) | 97(49.7%) | 46(38.0%) | 0.055 |
| BMI | 25.27±0.30 | 25.12±0.35 | 0.634 |
| **Coagulation function** |  |  |  |
| Fibrinogen | 3.45±0.05 | 3.21±0.09 | 0.021 |
| Prothrombin time | 11.30±0.13 | 11.30±0.20 | 0.989 |
| APTT | 26.87±0.30 | 27.06±0.42 | 0.701 |
| Thrombin time | 16.42±0.16 | 16.61±0.18 | 0.434 |
| INR | 0.99±0.02 | 0.97±0.02 | 0.267 |
| D-dimer | 0.38±0.04 | 0.65±0.16 | 0.043 |
| **Complication (%)** |  |  |  |
| DR (%) | 50(26.9%) | 42(39.3%) | 0.039 |
| DF (%) | 5(2.8%) | 2(1.9%) | 0.958 |
| DN (%) | 57(30.8%) | 33(31.7%) | 0.976 |
| PVD (%) | 16(8.9%) | 14(13.2%) | 0.341 |
| **MNSI exam** | 2.27±0.10 | 1.11±0.11 | <0.001 |
|  |  |  |  |
| Notes: Data are means ± SEM or n (%). DPN diabetic peripheral neuropathy group, T2DM diabetes mellitus with no peripheral neuropathy group, BMI body mass index, APTT activated partial thromboplastin time, INR international normalized ratio, DR Diabetic complication including diabetic retinopathy, DN diabetic nephropathy, DF diabetic foot, PVD peripheral vascular disease in type 2 diabetes mellitus, MNSI Michigan Neuropathy Screening Instrument. | | | |

| **Supplementary Table 2 -Multiple linear regression analysis of the correlation between coagulation function and electromyography parameters in the external validation (N=317).** | | | | | | | | | | | | | |
| --- | --- | --- | --- | --- | --- | --- | --- | --- | --- | --- | --- | --- | --- |
|  | **PT** | | **TT** | | **APTT** | | **INR** | | **D-dimer** | | **FIB** | |  |
| **Electromyography parameters β** | | ***P* value** | **β** | ***P* value** | **β** | ***P* value** | **β** | ***P* value** | **β** | ***P* value** | **β** | ***P* value** |  |
| **Motor NCV (m/s)** |  |  |  |  |  |  |  |  |  |  |  |  |  |
| Ulnar | -0.397 | 0.034 | -0.561 | 0.068 | -0.092 | 0.333 | -5.151 | 0.235 | -1.211 | 0.081 | -0.971 | <0.001 |  |
| Median | -0.216 | 0.226 | -0.589 | 0.043 | -0.142 | 0.113 | 1.991 | 0.632 | -1.426 | 0.025 | -1.204 | <0.001 |  |
| Peroneal | -0.172 | 0.256 | -0.782 | <0.001 | -0.046 | 0.551 | -3.158 | 0.373 | -0.444 | 0.420 | -0.424 | 0.068 |  |
| Tibial | -0.427 | 0.005 | -0.770 | <0.001 | -0.073 | 0.350 | -4.192 | 0.241 | -1.175 | 0.033 | -0.666 | 0.004 |  |
| **Sensory NCV (m/s)** |  |  |  |  |  |  |  |  |  |  |  |  |  |
| Ulnar | -0.070 | 0.696 | -0.558 | 0.065 | -0.169 | 0.061 | -5.108 | 0.227 | -0.206 | 0.762 | -0.615 | 0.027 |  |
| Median | -0.439 | 0.068 | 0.254 | 0.523 | -0.086 | 0.478 | -4.080 | 0.468 | -0.346 | 0.672 | -0.747 | 0.040 |  |
| Sup Peroneal | -0.094 | 0.584 | -0.407 | 0.164 | 0.048 | 0.630 | 5.730 | 0.178 | 0.850 | 0.223 | -0.538 | 0.070 |  |
| **Motor amplitude values** |  |  |  |  |  |  |  |  |  |  |  |  |  |
| Ulnar | -0.17 | 0.167 | -0.019 | 0.925 | -0.071 | 0.252 | -1.949 | 0.498 | -1.322 | 0.014 | -0.782 | <0.001 |  |
| Median | -0.157 | 0.160 | 0.135 | 0.458 | -0.083 | 0.139 | -1.930 | 0.460 | -1.258 | <0.001 | -0.622 | <0.001 |  |
| Peroneal | -0.121 | 0.316 | -0.231 | 0.221 | -0.017 | 0.785 | -0.116 | 0.967 | -0.971 | 0.030 | -0.534 | 0.004 |  |
| Tibial | -0.427 | 0.035 | -0.711 | 0.025 | -0.244 | 0.015 | -9.464 | 0.044 | -1.832 | 0.014 | -1.318 | <0.001 |  |
| **Sensory amplitude values** |  |  |  |  |  |  |  |  |  |  |  |  |  |
| Ulnar | -1.079 | 0.095 | -1.166 | 0.287 | -0.666 | 0.042 | -16.357 | 0.277 | -1.036 | 0.684 | -2.852 | 0.005 |  |
| Median | -0.770 | 0.228 | -1.506 | 0.155 | -0.375 | 0.258 | -7.727 | 0.606 | -2.635 | 0.292 | -2.864 | 0.003 |  |
| Sup Peroneal | -0.249 | 0.444 | -0.436 | 0.435 | -0.015 | 0.937 | -7.098 | 0.381 | 0.995 | 0.514 | -1.059 | 0.060 |  |
| **F-wave minimum latency(ms)** | 0.291 | 0.057 | 0.914 | <0.001 | 0.123 | 0.106 | 3.508 | 0.326 | 0.936 | 0.110 | 0.718 | 0.002 |  |
| **Mean velocity/amplitude** |  |  |  |  |  |  |  |  |  |  |  |  |  |
| MNAmp | -0.185 | 0.090 | -0.215 | 0.203 | -0.086 | 0.112 | -2.041 | 0.417 | -1.268 | 0.003 | -0.793 | <0.001 |  |
| MNCV | -0.270 | 0.060 | -0.673 | 0.002 | -0.066 | 0.357 | -1.886 | 0.569 | -0.994 | 0.049 | -0.830 | <0.001 |  |
| SNAmp | -0.506 | 0.276 | -0.860 | 0.251 | -0.317 | 0.174 | -3.387 | 0.751 | -1.443 | 0.429 | -2.664 | <0.001 |  |
| SNCV | -0.127 | 0.431 | -0.077 | 0.768 | -0.062 | 0.445 | 1.560 | 0.677 | -0.332 | 0.579 | -0.783 | <0.001 |  |
| **Summed Z scores** |  |  |  |  |  |  |  |  |  |  |  |  |  |
| amplitude Z score | -0.327 | 0.010 | -0.336 | 0.137 | -0.060 | 0.435 | -3.686 | 0.248 | -0.563 | 0.271 | -0.705 | 0.004 |  |
| velocity Z score | -0.328 | 0.021 | -0.584 | 0.019 | -0.038 | 0.649 | -1.292 | 0.718 | 0.377 | 0.491 | -0.444 | 0.102 |  |
|  |  |  |  |  |  |  |  |  |  |  |  |  |  |

Notes: PT Prothrombin time, TT Thrombin time, APTT activated partial thromboplastin time, INR international normalized ratio, FIB fibrinogen, NCV nerve conduction velocity, MNAmp mean motor nerve amplitude, MNCV mean motor nerve conduction velocity, SNAmp mean sensory nerve amplitude, SNCV mean sensory nerve conduction velocity.

#Adjusted for age, course of diabetes, hypertension, hyperlipidemia, BMI, smoking, FPG, HbA1c, TG, TC and CRP.

| **Supplementary Table 3- The presence of DPN and parameters of electromyography by tertiles (T1–T3) of FIB for each age group(N=849).** | | | | | | | | | | | | |
| --- | --- | --- | --- | --- | --- | --- | --- | --- | --- | --- | --- | --- |
| **Patients** | **Age<55** | | | | **55≤Age≤65** | | | | **Age>65** | | | |
| **FIB groups** | **T1** | **T2** | **T3** | ***P* value** | **T1** | **T2** | **T3** | ***P* value** | **T1** | **T2** | **T3** | ***P* value** |
|  | **<3.11** | **3.11-3.96** | **>3.96** |  | **<3.11** | **3.11-3.96** | **>3.96** |  | **<3.11** | **3.11-3.96** | **>3.96** |  |
| **Number** | 125 | 93 | 63 |  | 89 | 115 | 99 |  | 76 | 105 | 129 |  |
| **Age** | 44.42±0.63 | 45.45±0.77 | 47.52±0.71 | 0.011 | 59.50±0.33 | 59.74±0.30 | 59.67±0.30 | 0.852 | 71.10±0.55 | 71.72±0.43 | 72.41±0.46 | 0.174 |
| **FIB** | 2.64±0.03 | 3.46±0.02 | 5.15±0.14 | <0.001 | 2.68±0.04 | 3.48±0.02 | 5.10±0.10 | <0.001 | 2.60±0.05 | 3.52±0.02 | 5.29±0.11 | <0.001 |
| **DPN%** | 76(47.8%) | 56(50.9%) | 53(62.4%) | 0.183 | 60(54.5%) | 76(59.8%) | 78(67.8%) | 0.216 | 44(62.0%) | 74(67.3%) | 97(69.3%) | 0.843 |
| **Motor NCV (m/s)** |  | | | |  | | | |  | | | |
| Ulnar | 52.54±0.50 | 53.16±0.75 | 49.50±0.78 | <0.001 | 51.55±0.60 | 51.88±0.58 | 50.68±0.68 | 0.363 | 51.25±0.52 | 51.68±0.65 | 48.75±0.55 | <0.001 |
| Median | 53.58±0.36 | 53.32±0.61 | 51.10±0.74 | 0.004 | 52.77±0.62 | 52.75±0.60 | 50.99±0.51 | 0.050 | 52.41±0.55 | 52.15±0.44 | 50.39±0.43 | 0.003 |
| Peroneal | 44.37±0.43 | 44.55±0.56 | 41.01±0.64 | <0.001 | 43.94±0.51 | 43.90±0.46 | 43.32±0.52 | 0.628 | 43.21±0.60 | 43.23±0.40 | 41.35±0.38 | <0.001 |
| Tibial | 45.30±0.47 | 45.44±0.57 | 43.20±0.56 | 0.012 | 44.29±0.50 | 44.29±0.41 | 43.23±0.47 | 0.173 | 43.30±0.55 | 43.30±0.40 | 41.38±0.42 | <0.001 |
| **Sensory NCV (m/s)** |  | | | |  | | | |  | | | |
| Ulnar | 53.28±0.46 | 54.01±0.67 | 52.72±0.75 | 0.378 | 51.84±0.66 | 53.22±0.54 | 50.86±0.64 | 0.026 | 50.67±0.74 | 51.46±0.60 | 49.62±0.55 | 0.080 |
| Median | 54.21±0.57 | 53.80±0.75 | 51.02±0.87 | 0.008 | 52.58±0.66 | 52.12±0.64 | 49.95±0.79 | 0.020 | 49.25±1.00 | 49.96±0.81 | 48.03±0.67 | 0.186 |
| Sup Peroneal | 46.32±0.44 | 44.89±0.61 | 44.64±0.71 | 0.061 | 45.43±0.49 | 45.33±0.50 | 44.81±0.48 | 0.658 | 45.20±0.66 | 44.83±0.53 | 43.18±0.52 | 0.025 |
| **Motor amplitude values** |  | | | |  | | | |  | | | |
| Ulnar | 13.40±0.24 | 12.98±0.35 | 11.86±0.42 | 0.005 | 12.85±0.43 | 11.92±0.24 | 11.44±0.29 | 0.009 | 12.23±0.38 | 11.74±0.26 | 10.95±0.31 | 0.020 |
| Median | 13.74±0.27 | 14.00±0.33 | 12.72±0.37 | 0.030 | 12.75±0.31 | 12.39±0.29 | 10.75±0.29 | <0.001 | 11.03±0.31 | 10.89±0.44 | 10.09±0.25 | 0.094 |
| Peroneal | 8.56±0.33 | 8.08±0.42 | 6.24±0.41 | <0.001 | 7.38±0.57 | 6.42±0.29 | 5.36±0.32 | 0.003 | 6.28±0.42 | 5.07±0.30 | 4.54±0.27 | <0.001 |
| Tibial | 16.96±0.48 | 14.49±0.64 | 12.40±0.57 | <0.001 | 13.58±0.57 | 13.35±0.56 | 11.29±0.56 | 0.008 | 12.32±0.62 | 11.78±0.53 | 11.78±0.53 | 0.005 |
| **Sensory amplitude values** |  | | | |  | | | |  | | | |
| Ulnar | 41.01±1.89 | 44.02±2.07 | 36.62±3.05 | 0.118 | 34.14±1.80 | 34.93±1.79 | 30.14±2.21 | 0.179 | 31.58±2.26 | 29.80±1.83 | 26.97±1.72 | 0.234 |
| Median | 43.38±2.05 | 45.25±2.43 | 36.21±3.09 | 0.054 | 36.44±1.79 | 35.69±1.55 | 29.01±1.76 | 0.004 | 29.33±2.18 | 31.29±1.59 | 27.36±1.51 | 0.222 |
| Sup Peroneal | 15.96±1.13 | 16.06±1.60 | 13.01±1.05 | 0.302 | 12.45±0.77 | 12.21±0.64 | 11.60±0.75 | 0.707 | 11.90±0.97 | 10.24±0.56 | 9.58±0.50 | 0.050 |
| **F-wave minimum latency(ms)** | 44.16±0.33 | 44.16±0.52 | 46.44±0.61 | <0.001 | 44.79±0.45 | 45.08±0.45 | 45.72±0.46 | 0.346 | 45.26±0.61 | 44.61±0.44 | 47.29±0.44 | <0.001 |
| **Mean velocity/amplitude** |  |  |  |  |  |  |  |  |  |  |  |  |
| MNAmp | 13.14±0.24 | 12.23±0.32 | 10.63±0.35 | <0.001 | 11.81±0.47 | 10.83±0.26 | 9.58±0.29 | <0.001 | 10.30±0.33 | 9.75±0.27 | 8.80±0.26 | <0.001 |
| MNCV | 48.66±0.36 | 48.51±0.56 | 45.82±0.57 | 0.002 | 47.83±0.51 | 47.63±0.42 | 46.62±0.46 | 0.148 | 47.09±0.50 | 47.23±0.39 | 45.32±0.36 | <0.001 |
| SNAmp | 32.17±1.34 | 33.51±1.65 | 26.69±1.94 | 0.018 | 26.32±1.29 | 26.16±1.10 | 21.65±1.25 | 0.009 | 23.46±1.43 | 22.55±1.08 | 20.55±1.03 | 0.186 |
| SNCV | 50.95±0.38 | 50.26±0.58 | 48.95±0.62 | 0.025 | 49.81±0.46 | 49.75±0.44 | 48.14±0.48 | 0.016 | 48.06±0.61 | 48.28±0.49 | 46.70±0.43 | 0.035 |
| **Summed Z scores** |  |  |  |  |  |  |  |  |  |  |  |  |
| amplitude Z score | 3.42±0.38 | 3.39±0.45 | 2.29±0.58 | 0.196 | 0.97±0.39 | 0.66±0.33 | 0.28±0.43 | 0.071 | 0.27±0.48 | 1.19±0.37 | 1.51±0.39 | 0.120 |
| velocity Z score | 2.27±0.40 | 2.52±0.59 | 0.86±0.65 | 0.146 | 1.17±0.46 | 1.52±0.46 | 1.04±0.49 | 0.749 | 0.29±0.59 | 0.49±0.46 | 1.35±0.44 | 0.010 |
|  |  |  |  |  |  |  |  |  |  |  |  |  |

Notes: Data are means ± SEM or n (%). FIB plasma fibrinogen, DPN diabetic peripheral neuropathy group, NCV nerve conduction velocity, MNAmp mean motor nerve amplitude, MNCV mean motor nerve conduction velocity, SNAmp mean sensory nerve amplitude, SNCV mean sensory nerve conduction velocity. T1, tertile 1 of FIB; T2, tertile 2 of FIB; T3, tertile 3 of FIB.

| **Supplementary Table 4-Correlation analysis of FIB with electromyography parameters at each age group.** | | | | | | | | |
| --- | --- | --- | --- | --- | --- | --- | --- | --- |
| **Electromyography** | **Total(n=894)** | | **Age<55(n=290)** | | **55≤Age≤65(n=313)** | | **Age>65(n=291)** | |
| **parameters** | **r** | ***P* value** | **r** | ***P* value** | **r** | ***P* value** | **r** | ***P* value** |
| **Motor NCV (m/s)** |  |  |  |  |  |  |  |  |
| Ulnar | -0.195 | <0.001 | -0.195 | <0.001 | -0.142 | 0.013 | -0.196 | <0.001 |
| Median | -0.204 | <0.001 | -0.192 | <0.001 | -0.161 | 0.005 | -0.222 | <0.001 |
| Peroneal | -0.182 | <0.001 | -0.227 | <0.001 | -0.118 | 0.002 | -0.163 | 0.005 |
| Tibial | -0.177 | <0.001 | -0.168 | 0.002 | -0.119 | 0.028 | -0.164 | 0.004 |
| **Sensory NCV (m/s)** |  |  |  |  |  |  |  |  |
| Ulnar | -0.149 | <0.001 | -0.099 | 0.082 | -0.127 | 0.027 | -0.127 | 0.035 |
| Median | -0.198 | <0.001 | -0.185 | <0.001 | -0.167 | 0.004 | -0.161 | 0.008 |
| Sup Peroneal | -0.102 | 0.003 | -0.053 | 0.363 | -0.064 | 0.277 | -0.129 | 0.034 |
| **Motor amplitude values** |  |  |  |  |  |  |  |  |
| Ulnar | -0.187 | <0.001 | -0.121 | 0.031 | -0.219 | <0.001 | -0.148 | 0.013 |
| Median | -0.219 | <0.001 | -0.097 | 0.084 | -0.323 | <0.001 | -0.101 | 0.089 |
| Peroneal | -0.250 | <0.001 | -0.186 | <0.001 | -0.286 | <0.001 | -0.176 | 0.002 |
| Tibial | -0.268 | <0.001 | -0.282 | <0.001 | -0.257 | <0.001 | -0.164 | 0.004 |
| **Sensory amplitude values** |  |  |  |  |  |  |  |  |
| Ulnar | -0.172 | <0.001 | -0.101 | 0.077 | -0.181 | 0.002 | -0.136 | 0.025 |
| Median | -0.209 | <0.001 | -0.124 | 0.030 | -0.260 | <0.001 | -0.161 | 0.008 |
| Sup Peroneal | -0.102 | 0.003 | -0.009 | 0.881 | -0.124 | 0.035 | -0.143 | 0.024 |
| **F-wave minimum latency(ms)** | 0.187 | <0.001 | 0.177 | <0.001 | 0.137 | 0.012 | 0.205 | <0.001 |
| **Mean** **velocity/amplitude** |  |  |  |  |  |  |  |  |
| MNAmp | -0.295 | <0.001 | -0.262 | <0.001 | -0.336 | <0.001 | -0.165 | 0.003 |
| MNCV | -0.202 | <0.001 | -0.224 | <0.001 | -0.157 | <0.001 | -0.172 | 0.002 |
| SNAmp | -0.201 | <0.001 | -0.123 | 0.023 | -0.241 | <0.001 | -0.140 | 0.014 |
| SNCV | -0.191 | <0.001 | -0.151 | 0.005 | -0.177 | <0.001 | -0.149 | 0.009 |
| **Summed Z scores** |  |  |  |  |  |  |  |  |
| amplitude Z score | -0.204 | <0.001 | -0.050 | 0.420 | -0.215 | <0.001 | -0.165 | 0.016 |
| velocity Z score | -0.136 | <0.001 | -0.075 | 0.223 | -0.039 | 0.548 | -0.166 | 0.015 |
|  |  |  |  |  |  |  |  |  |

Notes: FIB plasma fibrinogen, NCV nerve conduction velocity, MNAmp mean motor nerve amplitude, MNCV mean motor nerve conduction velocity, SNAmp mean sensory nerve amplitude, SNCV mean sensory nerve conduction velocity
